# Amiodarone-Induced Hypothyroidism Related to Pericardial Effusion With Tamponade Physiology

**DOI:** 10.7759/cureus.22932

**Published:** 2022-03-07

**Authors:** Jennifer L Abrams, Daniel Fermi, Pooja Belligund, Samy I. McFarlane

**Affiliations:** 1 Internal Medicine, State University of New York Downstate Medical Center, Brooklyn, USA; 2 Pulmonary Critical Care, Veterans Affairs New York Harbor Healthcare System, Brooklyn Campus, Brooklyn, USA; 3 Internal Medicine: Diabetes and Endocrinology, State University of New York Downstate Medical Center, Brooklyn, USA

**Keywords:** pocus (point of care ultrasound, tamponade physiology, pericardial effusion, amiodarone, hypothyroidism

## Abstract

Hypothyroidism is a commonly encountered clinical diagnosis, particularly in the elderly population. While management of this disorder is rather simple with thyroxine replacement, healthcare providers may occasionally encounter patient non-adherence, which may lead to life-threatening complications. In this report, we present a case of a 74-year-old veteran with a long-standing history of amiodarone-induced asymptomatic hypothyroidism, who was non-adherent to thyroxine replacement therapy and presented to the hospital after a mechanical fall. His chest X-ray showed a globular heart with an enlarged cardiac silhouette, and transthoracic echocardiography (TTE) subsequently confirmed a large pericardial effusion with tamponade physiology. Physicians should be aware of and patients should be counseled about the potentially serious consequences of untreated hypothyroidism that could be avoided with proper patient education and adherence to the therapeutic plan.

## Introduction

As the global population ages, the incidence of hypothyroidism has been on the rise and is estimated to be at 7-14% among the elderly, most commonly due to autoimmune thyroiditis [1]. While many signs and symptoms of hypothyroidism are nonspecific and coincide with those related to aging, the severity of signs and symptoms may be exacerbated by certain comorbidities that are common among elderly patients [1]. Additionally, the incidence of severe complications of hypothyroidism, including myxedema coma, is much higher among the elderly [1]. Pericardial effusion is one such serious complication of hypothyroidism, with an incidence of about 3-6%, while the development of cardiac tamponade is less frequently encountered [2].

## Case presentation

A 74-year-old man with a history of hypothyroidism diagnosed in May 2014 and non-adherent to thyroxine replacement therapy, who had end-stage renal disease secondary to diabetes mellitus on hemodialysis since September 2020, non-ischemic dilated cardiomyopathy with recovered ejection fraction, and a history of ventricular tachycardia status post automated internal cardiac defibrillator and initiation of amiodarone in December 2013 presented after a mechanical fall. The patient was largely asymptomatic and did not report cold intolerance, weight changes, shortness of breath, chest pain, memory changes, muscle aches, or constipation. On physical examination, he did not exhibit classic features of hypothyroidism such as puffy eyes, dry skin, or macroglossia [2]. His initial vitals showed a pulse rate of 66 beats per minute and BP of 160/77 mmHg; his mental status was intact and he was alert and oriented to time, place, and person. However, a routine chest X-ray showed an enlarged cardiac silhouette concerning for possible pericardial effusion, which was confirmed on point-of-care ultrasound (POCUS) with evidence of tamponade physiology. Upon chart review, it was revealed that the patient’s hypothyroidism was likely induced by amiodarone, with abnormal thyroid function tests emerging five months after the initiation of the antiarrhythmic drug. Initial labs showed blood urea nitrogen (BUN) of 22 mg/dL, creatinine (Cr) of 4.6 mg/dL, sodium (Na) of 135 mEq/L, potassium (K) of 4.6 mEq/L, white blood cell count (WBC) of 5.4 x 10^9^/L, hemoglobin (Hb) level of 12.5 g/dL, hematocrit (HCT) of 39.1%, and platelet count of 273 x 10^3^/µl. Thyroid function tests over the preceding several months had demonstrated periods of non-adherence to levothyroxine therapy (Table 1).

**Table 1:** Thyroid function tests TSH: thyroid-stimulating hormone

	11/9/21	11/2/21	10/12/21	9/14/21	5/18/21	4/27/21	1/28/21	12/7/20	9/15/20
TSH, mU/L	82.194	90.279	35.675	64.351	13.368	20.681	6.013	3.216	26.44
Free T4, ng/dL	0.86	0.66	1.11	0.93	n/a	1.11	n/a	n/a	1.22
Levothyroxine dose	200 mcg	200 mcg	200 mcg	200 mcg	200 mg	200 mcg	200 mcg	200 mcg	200 mcg

**Figure 1:**
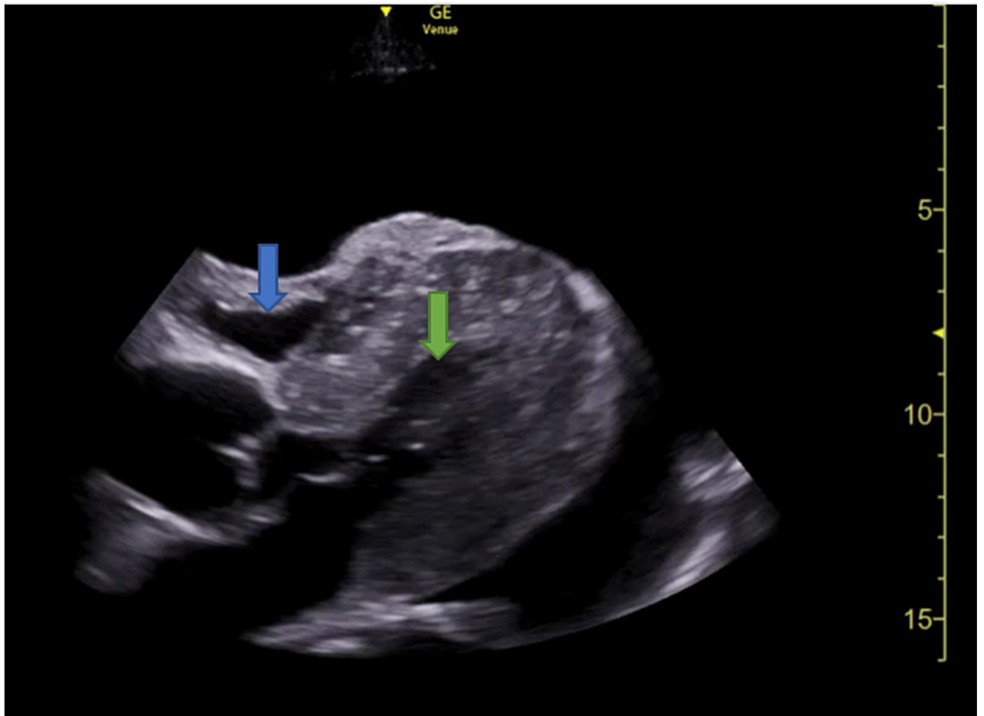
Echocardiography showing right ventricular collapse during diastole Blue arrow: right ventricle. Green arrow: left ventricle

The patient's EKG showed atrial fibrillation with low-voltage QRS. Given the size of the effusion, dialysis was halted due to concerns about worsening tamponade physiology. Cardiology was consulted and a formal transthoracic echocardiogram (TTE) was obtained with confirmation of pericardial effusion with tamponade (Figure 1). The increased pericardial pressure from the effusion caused the collapse of the right ventricle during diastole.

**Figure 2:**
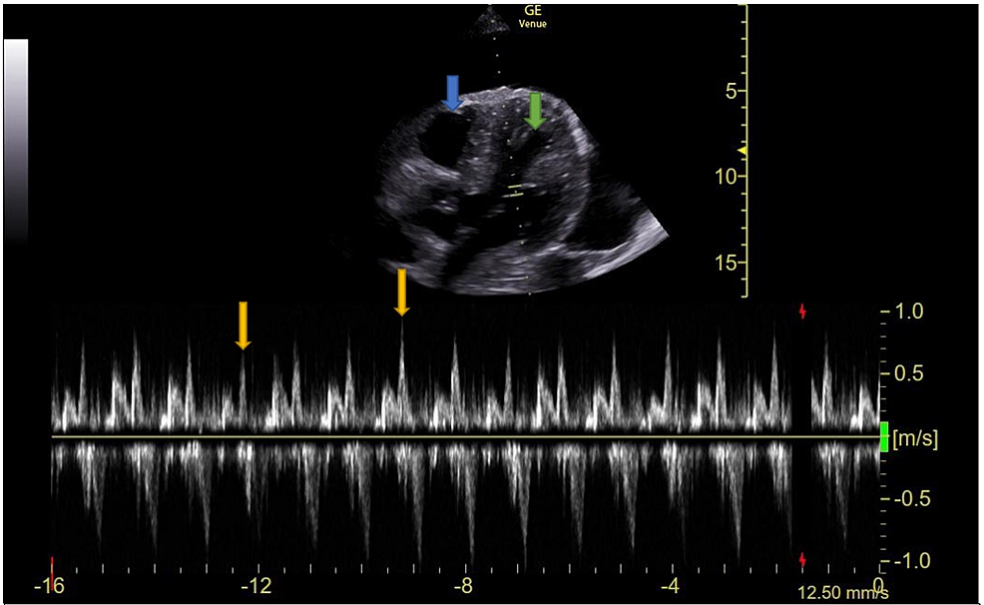
Echocardiography with pulse wave Doppler at the mitral valve showing ventricular interdependence, concerning for tamponade physiology Orange arrows show greater than 25% variability of passive filling of the left ventricle during the respiratory cycle. Blue arrow: right ventricle. Green arrow: left ventricle. Yellow brackets: mitral valve

Pulse wave Doppler of the mitral valve inflow was obtained, which showed greater than 25% variability of the rapid filling (E wave) during inspiration (Figure 2). This finding was concerning for tamponade physiology and indicated decreased systolic blood pressure secondary to the right ventricle bowing into the left ventricle.

The patient was restarted on levothyroxine 200 mcg daily. He was subsequently transferred to a sister facility where successful drainage of pericardiocentesis was performed with 1800-cc drainage. Pericardial fluid cultures were negative for any growth. The patient’s levothyroxine dose was slowly up-titrated to an appropriate dose to normalize his thyroid function. Amiodarone was stopped by Cardiology given the severe hypothyroidism. In addition, the patient was counseled extensively on the importance of medication compliance. He was discharged from the hospital to a subacute rehabilitation center with close outpatient follow-up with Cardiology and Endocrinology. However, upon discharge from the rehabilitation center, the patient again became non-adherent to medications. A repeat TTE one month later showed that the patient had re-accumulation of the pericardial effusion with tamponade physiology. At that time, the patient underwent pericardiocentesis with a surgical pericardial window as definitive treatment for his pericardial effusion.

## Discussion

The patient’s hypothyroidism was likely caused by amiodarone, an antiarrhythmic medication with several adverse effects. Notably, amiodarone has been found to cause hypothyroidism in about 5-25% of cases [3]. Amiodarone’s iodine-rich structure inhibits the peripheral conversion of T4 to T3. The low levels of T3 seen in the pituitary gland may lead to an elevation in the thyroid-stimulating hormone (TSH) and can result in overt hypothyroidism [3]. In addition, amiodarone has direct effects on the thyroid gland due to the Wolff-Chaikoff effect in which the presence of excess iodine inhibits iodine organification needed for thyroid hormone synthesis, leading to reduced production of the thyroid hormone [3]. Patients taking amiodarone should be monitored regularly for thyroid dysfunction and symptoms of hypo- or hyperthyroidism. The disease is confirmed by the presence of elevated TSH and low free T4 in the setting of amiodarone use. This patient was identified to have amiodarone-induced hypothyroidism soon after starting the antiarrhythmic medication and was started on thyroxine replacement therapy according to guidelines. However, his medication non-adherence contributed to uncontrolled hypothyroidism and increased his risk of complications. This ultimately led to the decision to discontinue amiodarone altogether.

There are several known cardiac complications of hypothyroidism, including EKG changes such as sinus bradycardia, low-amplitude QRS, prolonged QT, and altered T wave morphology [4]. This patient's EKG showed atrial fibrillation with low-voltage QRS, characterized by a QRS complex of less than 0.5 mV in frontal leads and less than 1 mV in precordial leads [2]. In addition, hypothyroidism causes pericardial effusion in about 3-37% of cases [5]. Hypothyroidism leads to increased vascular permeability to albumin. In the pericardial tissues, this leads to the accumulation of fluid causing pericardial effusion [5]. In severe hypothyroidism, this often leads to larger effusions, which put patients at the risk of cardiac tamponade. There have been very few reports of pericardial effusion in amiodarone-induced hypothyroidism, making this a seemingly rare complication. Echocardiography is the gold standard imaging technique to diagnose and quantify the size of pericardial effusion and to identify tamponade physiology [6]. In cardiac tamponade, external compression of the heart leads to impaired ventricular filling during diastole [6]. This can ultimately lead to hemodynamic instability, making prompt diagnosis and treatment crucial.

Long-standing severe hypothyroidism may additionally lead to myxedema coma, a medical emergency with high mortality rates. Typical myxedema coma is characterized by altered mental status, hypothermia, and a precipitating event [7]. In our case, the patient presented with a mechanical fall and severe biochemical hypothyroidism; however, he remained clinically euthyroid without any signs of myxedema coma. His overt biochemical hypothyroidism did, however, necessitate immediate treatment to prevent progression to myxedema coma. Guidelines recommend lower dosing of thyroxine replacement therapy in elderly patients (>65 years old), especially those with cardiovascular risk factors [8]. As people age, there is typically a decreased turnover of T4, leading to a lower level of levothyroxine needed to normalize TSH levels [8]. Avoidance of adverse effects, particularly atrial fibrillation and osteoporosis, is also of utmost importance in elderly populations.

In this case, the patient had a large pericardial effusion with tamponade physiology. The insidious onset of his symptoms likely points to long-standing effusion that allowed for compensation, and explains his asymptomatic presentation. This is typical of effusion associated with hypothyroidism. A sudden accumulation of fluid, such as during trauma, is more likely to cause symptoms even when there is a small collection of fluid [6]. This likely explains why the classic Beck’s triad - jugular venous distension, hypotension, and muffled heart sounds - was not present [6]. This patient’s scheduled hemodialysis session was immediately halted when his pericardial effusion was identified, as this fluid shift could lead to severely decreased preload and cardiac output, causing hemodynamic instability. Prompt drainage of the pericardial effusion is essential in patients exhibiting tamponade physiology, as seen in this patient.

## Conclusions

We presented a case of a 74-year-old man with amiodarone-induced asymptomatic hypothyroidism with a history of non-adherence to levothyroxine therapy with impending cardiac tamponade. Although our patient did not present with classic signs of severe hypothyroidism or myxedema coma, physicians should be alert to the possibility of a large pericardial effusion with impending tamponade that could be life-threatening in asymptomatic patients with a rather unrelated presentation. Regardless of the timeframe of symptom onset, patients with large pericardial effusions have the potential to decompensate rapidly, especially those with large volume shifts such as those undergoing hemodialysis, as was the case with this patient.
